# Image processing and recognition for biological images

**DOI:** 10.1111/dgd.12054

**Published:** 2013-04-07

**Authors:** Seiichi Uchida

**Affiliations:** Department of Advanced Information Technology, Kyushu University744 Motooka, Nishi, Fukuoka, 819-0395, Japan

**Keywords:** bioimage informatics, image pattern recognition, image processing

## Abstract

This paper reviews image processing and pattern recognition techniques, which will be useful to analyze bioimages. Although this paper does not provide their technical details, it will be possible to grasp their main tasks and typical tools to handle the tasks. Image processing is a large research area to improve the visibility of an input image and acquire some valuable information from it. As the main tasks of image processing, this paper introduces gray-level transformation, binarization, image filtering, image segmentation, visual object tracking, optical flow and image registration. Image pattern recognition is the technique to classify an input image into one of the predefined classes and also has a large research area. This paper overviews its two main modules, that is, feature extraction module and classification module. Throughout the paper, it will be emphasized that bioimage is a very difficult target for even state-of-the-art image processing and pattern recognition techniques due to noises, deformations, etc. This paper is expected to be one tutorial guide to bridge biology and image processing researchers for their further collaboration to tackle such a difficult target.

## Introduction

Development of imaging techniques, especially, microscopic imaging techniques, has made it possible to visualize various biological phenomena for biologists. Imaging results are provided as still-images or videos. In more elaborate cases, 3D volumetric images and 4D volumetric videos are provided. Behavior and distribution of molecules are also visualized by fluorescence technologies. Biologists can observe those imaging results, that is, bioimages, for their further analyses of biological phenomena.

Biologists often perform manual inspections for bioimage analysis. For example, a biologist might track an organelle by watching all video frames by his/her eyes for analyzing its movement in a cell. It will require a large effort and concentration. If there are multiple target organelles, it requires far larger efforts. Consequently, it is difficult to analyze a huge number of bioimages and to have reliable analysis results. Furthermore, we need to be careful that the analysis result by the manual inspection may be biased by subjective observation. That is, the analysis result will depend largely on personal skill, decision, and preference.

On the other hand, research on “bioimage-informatics” is becoming active. The purpose of bioimage-informatics is to analyze bioimages automatically or semi-automatically by computer. The techniques for bioimage-informatics will be very helpful to biologists to deal with a large number of bioimages and exclude subjective biases. If the techniques are accurate enough, their analysis results will be more accurate and reliable than those by manual inspection. Although bioimage-informatics has a rather long history and thus it is easy to find many past trials especially on blood cell segmentation and cell counting (such as Parthenis *et al*. 1989), recent progress in microscopic technologies is enhancing the research activity. This can be understood by several survey papers (Peng 2008; Shamir *et al*. 2010; Danuser 2011; Kanade *et al*. 2011; Schneider *et al*. 2012) and the special issue of *Nature Methods* from July 2012, on the topic of bioimage-informatics.

This paper introduces several basic image processing and image pattern recognition techniques, which will be useful for analyzing bioimages automatically by computer. This paper also can be used for a tutorial guide in advanced use of image processing software (Swedlow *et al*. 2009; Schneider *et al*. 2012), such as ImageJ, Fiji, OpenCV, Matlab, R, and MetaMorph, and pattern recognition software (Shamir *et al*. 2010). Note that image compression, computer graphics, and computational photography are also important fields of image processing, although they are not covered by this review paper.

Since this paper mainly outlines major techniques for bioimage-informatics, it does not explain their individual details, such as their mathematical principles. Readers who want to know more details of those conventional techniques or to implement a certain method or to learn more sophisticated methods, are better to refer to the following text books or survey papers:

Image processing

A famous image processing text book including gray-level transformation, binarization, filtering, color, mathematical morphology, and image segmentation (Gonzales & Woods 2007).
A classical text book but wide topics, including filtering, image segmentation, texture, optical flow, and also pattern recognition (Ballard & Brown 1982).^1^
A survey paper on recent image filtering techniques (Milanfar 2013).
A survey paper on visual object tracking (Yilmaz *et al*. 2006).
A survey paper on optical flow (Beauchemin & Barron 1995).
Survey papers on image registration (McInerney & Terzopoulos 1996; Glasbey & Mardia 1998; Jain *et al*. 1998; Redert *et al*. 1999; Uchida & Sakoe 2005).
A survey paper on local features for image registration and tracking (Mikolajczyk & Schmid 2005).

Pattern recognition

Two most famous books on the fundamentals of pattern recognition (Duda *et al*. 2000; Bishop 2006).
A survey paper on clustering (Jain *et al*. 1999).

## Overview of image processing and pattern recognition

One of main purposes of image processing is to manipulate pixel values for better visibility. For example, gray-level transformation and image filtering are typical image processing techniques for converting an input image into a new image with better visibility. Another purpose of image processing is to extract some target objects or regions from an input image. For example, if we extract all organelles of a specific kind, we can count them and also understand their distribution and behavior in a cell.

On the other hand, a general purpose of image pattern recognition is to classify an image or a target object or a region into one of types, i.e. classes. Although it is difficult to achieve the recognition accuracy of human beings, image pattern recognition has already been used in various applications. Optical character recognition (OCR) is one of the most classic applications, where an image of a single character is classified into one of 52 classes (“A”–“Z”, “a”–“z”). Recognition of faces and more general visual objects (such as car, chair, and daily goods) is also a very active recent research topic. Note that we sometimes encounter cases where classes are not predefined. For example, in order to recognize leaf types, we need to define shape types as classes. In this case, we will start making several groups from all images and then a class name is assigned to each group. This grouping technique is called clustering.

Table 1 indicates how to select image processing and recognition techniques according to our purpose. All of those techniques can be applicable to biological image analysis. Note that there is no strict boundary between image processing and image pattern recognition. Many intelligent image processing techniques rely on pattern recognition techniques. Pattern recognition techniques also use image preprocessing techniques for extracting important discriminative information (called features) from a target image.

**Table 1:** Image processing and recognition methods which fit to a specific purpose

What do you want to do?		Method	Note
I want to improve visibility of my image by…	controlling contrast	Gray level transform	It is also possible to calibrate gray levels of two images via gray-level transformation
		Binarization	Appropriate for essentially (or approximately) binary images
	removing noise	Image filtering	Smoothing filters
	emphasizing object boundary		Edge detection filters, sharpening filters
I want to extract target objects in my image for…	counting them, understanding their boundaries, evaluating their shape and size	Binarization	Simple but applicable only when the target objects have only brighter (or darker) pixels than the background
		Image segmentation	Image recognition techniques are also often used
I want to analyze the motion in my video by…	tracking a single or multiple target objects	Visual object tracking	It is comprised of two sub-problems, i.e. detection of the target objects at each frame and connection of the detected results over frames to form temporal trajectories
	determining the motion in the entire image	Optical flow	It is possible to interpret optical flow as a set of tracking results of all pixels in the image
I want to compare two images by overlaying them flexibly, i.e. elastically		Image registration	Non-parametric image registration is mathematically similar to optical flow
I want to classify images or targets into several types		Image recognition	If classes are already defined
		Clustering	If classes are not defined in advance

**Fig. 1:**
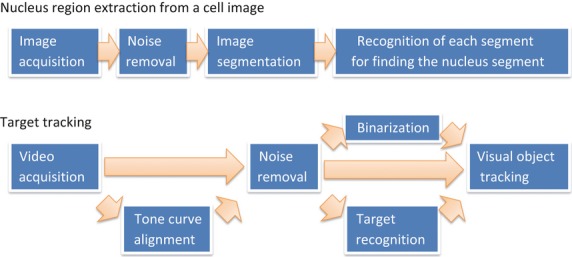
Combination of multiple image processing and image recognition techniques for realizing a complete system for a specific task.

It is rather rare to use a single image processing technique or a single image pattern recognition technique. In fact, they are often used in a mixed manner in order to realize a complete system for a specific task. For example, for extracting target organelles from an image, an image segmentation technique is first applied to the image and then each segment (i.e. region) is fed into an image recognition technique for deciding whether the segment is target or not. Figure 1 shows other examples. Consequently, we need to understand the function of individual techniques and useful combinations of the techniques.

It is very important to understand the fact that biological images are often far more difficult to be processed and recognized than popular (i.e. daily-life) images, such as character, face, and person images. In particular, microscopic bioimages have the following difficulties for image processing and recognition.

Difficulties from image acquisition conditions:

Noise: Since original light intensity from microscopes (especially, florescence microscopes) is weak, the acquired images are often noisy. Furthermore, florescence images are often contaminated by autofluorescence.
Low resolution.
Low frame rate.
Blur.

Difficulties from characteristics of target objects:

Less appearance information (just a bright spot or tiny object).
Unstable brightness (especially, due to the photobleaching phenomena and dying cells).
Multiple targets.
Overlapping targets.
Transparent objects.
Unclear boundary/texture.
Severe deformation (e.g. dying cells and elastic organelle).
Lack of motion and deformation models.

Due to the above difficulties, even state-of-the-art image processing and recognition methods may suffer from difficulties in analyzing biological images.

## Gray-level transformation and binarization

A digital image is comprised of a finite set of pixels, which are arranged in two dimensional plain. A pixel has a pixel value. For gray-scale image, a pixel has an integer value between 0 and 255. This is so called an 8-bit pixel-value since an 8-bit value can represent 2^8^ = 256 different values, i.e. 0–255. In this paper, any gray-scale image is assumed to have 8-bit pixel values, unless otherwise mentioned. In several applications, we also can use *n*-bit images, where *n* is larger or smaller than 8, for representing 2^*n*^ different values. For color images, a pixel typically has a three-dimensional integer vector, for example, (120, 80, 5), where each vector element represents red, or green, or blue values. Each component value lies, usually, between 0 and 255. This is so-called 24-bit RBG color image (24 = 8 × 3[RGB]). In this paper, any color image is assumed to be a 24-bt RGB color image, unless otherwise mentioned. Note that there are many variations in color image representation (Gonzales & Woods 2007). Although RGB is a typical color model, Hue-Saturation-Value (HSV) has often been used for expecting its robustness to intensity change after disregarding the V component.

### Contrast enhancement

**Fig. 2:**
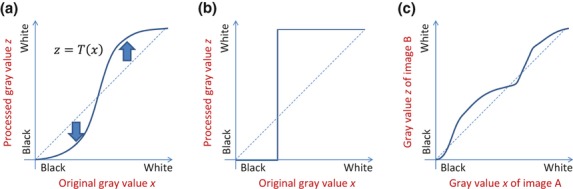
Gray-level transformation by tone curve *z* = *T*(*x*). (a) Gray-level transformation for contrast enhancement. (b) Binarization as a gray-level transformation. (c) Tone curve alignment between a pair of images.

Gray-level transformation is an image processing technique to convert a gray-level value to another value. A simple example is the “negative” processing where gray-level value 0 is converted to 255, 255 to 0, and, generally, *x* to 255−*x*, for a gray-scale image with the gray-level range from 0 to 255. This conversion is represented as a mapping function *z* = *T*(*x*), where *x* and *z* are the original and the converted pixel values. The negative processing is represented as *z* = *T*(*x*) = 255−*x*. This mapping function is called tone curve. Figure 2a illustrates a tone curve. By changing the shape of the tone curve, it is possible to realize various gray-level transformations.

Figure 2a shows a tone curve for image enhancement. This is because this curve converts dark gray values to darker, and bright ones brighter (as indicated by arrows) and consequently, the difference between dark regions and bright regions becomes more apparent. The detailed shape of the tone curve should be designed by observing the resulting image.

Figure 2b shows an extreme case of gray-level transformation for image enhancement. This tone curve performs image binarization, where any pixel brighter than a threshold becomes white and lower becomes black. The position *x* of the jump in the tone curve corresponds to the threshold. Note that binarization methods are detailed later.

### Tone curve alignment

When we process two similar images captured at different timings, we sometimes encounter a non-negligible difference in their brightness. This difference may happen due to automatic aperture and/or shutter control during image acquisition. Usually, it is better to remove such a difference in advance to further processes; without the removal, a parameter value suitable for image A is not suitable for image B. In addition, there is a risk that they are recognized as very different images just because of their brightness difference.

Tone curve alignment is a technique to remove the brightness difference as much as possible by using a tone curve determined automatically and optimally. Consider gray-level histograms *H*_A_(*x*) and *H*_B_(*z*) for images A and B, respectively, where *x* and *z* are the pixel values of images A and B, respectively, and *H*_A_(*x*) counts the number of pixels having the pixel value *x*. An optimal tone curve *z* = *T*(*x*) to remove their brightness difference between A and B should minimize the difference in their gray-level histograms. In other words, the histogram of image B after gray-level transformation, i.e. *H*_B_(*T*(*x*)) should be similar to *H*_B_(*z*) as much as possible. Formally, this optimization problem is defined as:


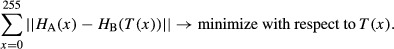


The remaining problem is how to find *T*(*x*) that minimizes the above closeness criterion. This tone-curve optimization problem can be seen as a “path” optimization on the two-dimensional *x*-*z* plane as shown in Figure 2c. (Mathematically, this is a kind of variational problem.) Although there are 256^256^ possibilities for the optimal curve, dynamic programming can provide the optimal path (i.e. tone curve) with about 256^2^ × *const* computations, where *const* is a positive constant. Dynamic programing is a method to drive an optimal solution to certain types of optimization problems very efficiently.

Note that one curve alignment should not be applied to bioimages whose original brightness values are meaningful. For example, if the target brightness is used for evaluating its copy number variation, tone curve alignment should not be applied.

### Binarization

Binarization is the operation to convert an original grayscale image into a black-and-white image and has multiple purposes. Separation of bright target objects from dark background (and *vice versa*) is a typical purpose. The resulting image contains connected black-pixel regions and also white-pixel regions. Each of those regions is called a connected component. By counting the white connected components, it is possible to count bright target objects. It is also possible to analyze the size and the shape of each target object by observing its corresponding connected component. For example, we can analyze the circularity of individual objects. In this sense, binarization can be considered as a kind of image segmentation method, which is detailed later.

As described below, there are three types of binarization methods: global thresholding, local thresholding, and optimization-based methods. Table 2 summarizes their merits and demerits.

**Table 2:** A list of binarization methods

Name	Methodology	Merit	Demerit
Global thresholding	First, a single threshold value is determined for the whole image. Then, the gray-value at each pixel is compared to the threshold. If the gray-value is larger (i.e. brighter), it is converted to white	Generally simple. Many variations for determining the threshold	Unsuccessful when the range of gray-scale varies locally (by, for example, uneven lighting condition and non-uniform background)
Local thresholding	For each pixel, a threshold value is determined by considering a surrounding region	Better performance where the global thresholding method will fail	Generally more computations. A special treatment is necessary for the case where the surrounding region is essentially uniform
Optimization-based binarization	A Markov random field (MRF) formulation where black/white decision is done at each pixel while considering the decisions at neighboring pixels	Using appropriate optimization scheme, the decision becomes more robust to gray-level fluctuations. Solid mathematical basis	Necessary to design the problem formulation as a mathematical optimization problem. Sometimes, computationally expensive

#### Global thresholding

Global thresholding is the binarization method that pixels with a gray-level value larger than a fixed threshold value are converted into white pixels and the others are black. The threshold value is the same for all pixels in the image. Thus, global thresholding assumes that the grayscale distributions of bright target objects and dark background are constant over the image.

Global thresholding methods can be characterized by their strategy to determine the threshold value. The simplest method is to determine the value manually. For automatic determination, the histogram *H*(*x*) of gray-scale pixel values plays an important role. The number of histogram bins is the number of possible gray-scale pixel values, say, 256. The *x*th bin of the histogram shows the number of pixels with the gray-scale pixel value *x*. If the histogram is bi-modal, that is, if the histogram has two peaks and a valley between them, we can set the threshold at the value of the valley. This is the so-called mode method.

The P-tile method is applicable if we (roughly) know the total number *P* of pixels that should be white. While accumulating the histogram value from the brightest bin to the darkest, the threshold value is determined at the bin value where the accumulated value exceeds *P*.

The Otsu's method is applicable to more general cases. It determines the threshold value that maximizes a criterion function that evaluates the separation of the given histogram by the threshold value. Simply speaking, the threshold is determined at the value that separates the histogram into two parts as clearly as possible. More specifically, the criterion function is designed to become larger when the mean values of both sides of the histogram separated by the threshold value are more different and the both sides have less variance. Accordingly, if the histogram is bi-modal, the threshold value is determined around the valley like the mode method. This idea is generally applicable to any histogram shape. For example, when the histogram is uniform between the brightest (255) and darkest (0) gray values, the threshold value is determined at the middle value, i.e. 128. The Otsu's method is based on discriminant analysis, which is a statistical analysis method.

In some cases it is useful to estimate the background noise level caused by imaging devices for determining the threshold value. In fluorescence observation, it is possible to estimate the background noise caused by autofluorescence using the brightness level of the cytoplasm of a control cell. These levels provide an important clue for fixing the global threshold value.

#### Local thresholding

**Fig. 3:**
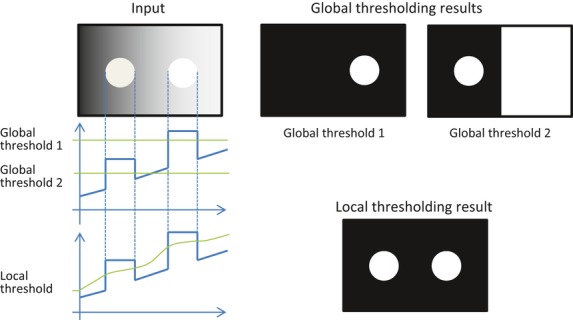
Binarization by global thresholding and local thresholding. For this input image, no global threshold provides the result where two circles are successfully extracted. In contrast, the local threshold determined at each pixel by the local average around the pixel will provide a successful result.

Sometimes, global thresholding is insufficient; that is, the constant threshold value over the image is insufficient. For example, it will happen that a part of the background region is brighter than some target region. In this case, global thresholding cannot extract all the targets without any false extraction from the background region. Figure 3 illustrates this insufficiency. The shaded background is partially brighter than a (darker) target and no global threshold can extract those two targets correctly.

Local thresholding will solve this problem by setting an appropriate binarization threshold at each pixel. For example, the Niblack's method sets the threshold value at a pixel *x* at the sum of an average pixel value in a small region around *x* and a small negative (or positive) value. Figure 3 shows the effect of local thresholding, which can avoid the above problem of global thresholding. The size of the small region and the small value are determined manually through preliminary experiments.

#### Optimization-based binarization

Binarization can be formulated as an optimization problem to decide whether each pixel belongs to one of two classes, white or black. The class decision will be done by considering two factors. The first factor is how the final decision (black and white) is similar to the original pixel value. In other words, the binarization result at a pixel *x* should be closer to the original pixel value of *x*. The second factor is that the binarization result at a pixel *x* is better to be the same as the results of the neighboring pixels of *x*. Without the second factor, this optimization is easily done by using global thresholding with the threshold value 128 for the grayscale image whose gray-level ranges from 0 to 255. Without the second factor, however, the binarized image often becomes noisy — small black (or white) regions are unnecessarily scattered over the image. Thus, the second factor is useful to make the binarization result smooth.

This formulation, where the decision at a pixel depends on the decisions of neighboring pixels, is called Markov random field (MRF). The binarization problem is now formulated as an optimal decision problem on MRF. The optimal decision i.e. the optimal binarization result for an *M* × *N* image is provided by using an optimization method that can find the best decision from 2^*MN*^ possible decisions. Clearly, it is practically impossible to evaluate all those possibilities in the one-by-one manner. We, therefore, have to resort to a sophisticated optimization method, which requires far less computation. Fortunately, the optimization method called graph cut can provide the optimal result with very practical computations (precisely, polynomial-order computations). A survey of the other optimization methods for MRF can be found in Szeliski *et al*. (2008).

## Image filtering

Image filtering is often the first step of image processing. By image filtering, an input image is converted to another image with a different property. For example, a smoothing filter converts an input image to its smoothed version, where small intensity (or color) difference between neighboring pixels is minimized.

Table 3 lists image filtering techniques according to their purpose, i.e. smoothing, edge detection, and image sharpening. Each technique will be detailed later. Image filtering techniques are roughly divided into two types: linear filters and nonlinear filters, according to the way of filtering operations. The linear filters are based on linear operations, such as addition and constant multiplication, whereas nonlinear filters are based on nonlinear operations, such as minimum value selection. Note that a linear filter and a nonlinear filter may have the same purpose. For example, there are linear smoothing filters and nonlinear smoothing filters.

**Table 3:** A list of image filtering methods

What do you want to do by filtering?	Filter name	Filter type	Note
Smoothing	Blurring filter	Linear	A kind of low-pass filters
	Median filter	Nonlinear	Good for removal of salt-pepper (dot) noise
	Bilateral filter	Nonlinear	Edge-preserving smoothing
Edge detection	Sobel filter	Linear	Calculate *X* and *Y* gradients independently
	Laplacian filter	Linear	Calculate two-dimensional gradient at once
	Canny filter	Nonlinear	Recently, one of the most popular image detection techniques
Sharpening	Unsharp masking	Linear	Emphasize non-smooth component
Reshape connected components in a binary image	Morphological filters	Nonlinear	For example, it is possible to fill small holes in a connected component and remove small connected components. Extendable to deal with gray-scale images

### Smoothing

**Fig. 4:**
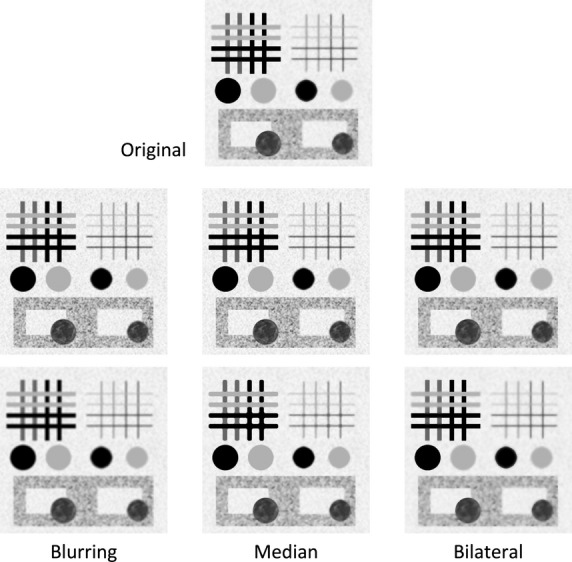
Effect of three different smoothing filters. Top: Original image. Middle: Mild effect. Bottom: Strong effect. The original image contains linear and textured components. Note that the components in the right side of the original image are blurred intentionally. For blurring and median filters, 3 × 3 mask (mild) and 7 × 7 mask (strong) were used. For bilateral filter, its filtering operation with a 7 × 7 Gaussian mask was done once (mild) and three times (strong). All of those filters are implemented in OpenCV.

Smoothing aims to minimize gray-level difference among neighboring pixels. The gray-level difference is often caused by noise and thus smoothing is useful for noise removal. There are three typical image filtering methods: blurring filter, median filter, and bilateral filter. Figure 4 shows the result of these image filters.

1. Blurring filter replaces the original pixel value by an average pixel value around the pixel. Since the averaging operation cancels noise, the resulting image becomes smooth. Consider a one-dimensional image (9, 12, 9, 10, 8, 10). If we take the average among a pixel and its two neighborhoods, the image becomes (10.5, 10.0, 10.3, 9.0, 9.3, 9.0). As shown by this example, the difference between the neighboring pixels can be minimized.
2. A median filter replaces the original pixel value by the median pixel value among the current pixel and its neighboring pixels. A median filter can remove salt-and-pepper noise, i.e. dot noise. Consider a one-dimensional image (0, 0, 100, 0, 1, 0), where 100 is an abrupt change of the pixel value and thus considered as a dot noise. If we take the median value among a pixel and its two neighborhoods, the image becomes (0, 0, 0, 1, 0, 0) and the dot noise is removed successfully. Note that by the blurring filter, it becomes (0, 33.3, 33.3, 33.6, 0.3, 0.5) and the dot noise spreads to the neighboring pixels.
3. A bilateral filter is a kind of edge-preserving smoothing method. The above two methods often smear edges as their side-effect of smoothing. In contrast, bilateral filter diminishes this side-effect by controlling the degree of smoothing according to the “edgeness.” If there is a big gray-scale difference around a pixel, the pixel is considered as an edge pixel and blurring effect around it is weakened.

Note that the above blurring filter is a typical linear filter and its filtered value is determined by inner-product, which is a linear operation. In the above example, the smoothed value 10.0 was determined by the inner-product between (9, 12, 9) and (1/3, 1/3, 1/3). Similarly, 10.3 was determined by the inner-product between (12, 9, 10) and (1/3, 1/3, 1/3). The vector (1/3, 1/3, 1/3) is called a filter mask. If we use a different filter mask, the filtered image becomes different. In other words, we can develop various linear filter techniques by changing the filter mask. For example, the mask (1/4, 1/2, 1/4) provides a weighted smoothing filter. If we use Gaussian function as a filter mask, we can have a Gaussian smoothing (or Gaussian blurring) filter.

### Edge detection

Edge detection filter is another popular filter. Edge is defined as a set of pixels with a large change in pixel value. For an image with a white-filled circle on a black background, the edge is a boundary of the circle. By edge detection filter, edge pixels are highlighted. The basic idea of edge detection filtering is to calculate the first-order or the second-order derivatives of pixel values.

**Fig. 5:**
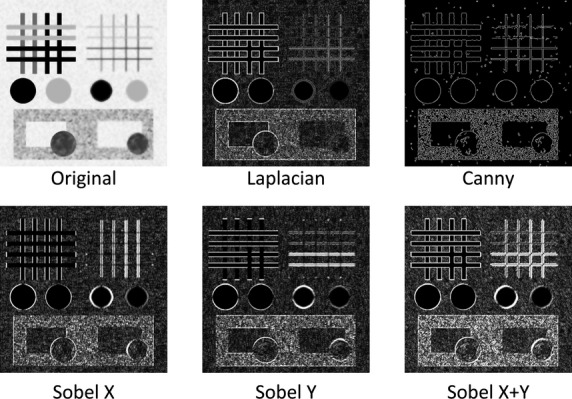
Effect of three different edge detection filters, Laplacian, Canny, and Sobel filters. Note that the components in the right side of the original image are blurred intentionally. Sobel filter detects vertical edges (Sobel X) and horizontal edges (Sobel Y) independently. They are added to have all edges (Sobel X + Y). All of those filters (except for Sobel X + Y) are implemented in OpenCV.

Like smoothing filters, there are many edge detection filters. Figure 5 shows the result of three different edge detection filters, called Laplacian, Canny, and Sobel filters. Sobel filter is one of the simplest edge detection filters and comprises the first-order *x*-derivative and *y*-derivative operators. Similarly, Laplacian filter is based on the second-order *x*-derivative and *y*-derivative operators. Sobel and Laplacian filters are linear filters and thus are represented by their own filter mask. Note that as observed in Figure 5, edge detection filters often emphasize noisy background unnecessarily. One remedy to suppress such noises is to apply some smoothing filter in advance of applying the edge detection filter. From a view point of signal processing, this combination use of two filters results in a band-pass filter, where the smoothing filter works as a low-pass filter and the edge detection filter as a high-pass filter.

Canny filter is a more sophisticated and popular technique because it is more robust to noise – it can suppress spurious edge pixels. Its result is a binary image where white pixels indicate edge pixels selected by not only having their large value in Sobel filter response but also satisfying local maximum conditions and connectivity to the local maximum.

A sharpening filter is an extension of the edge detection filter and can emphasize the edge region in an image. It can be defined by the sum of the original image and the Laplacian edge image. Instead of the Laplacian edge image, it is possible to use the subtraction of a smoothed image from its original image. This version is called unsharp masking.

## Image segmentation

Image segmentation is one of the most important image processing techniques for biological images. Its purpose is to partition an input image into regions. Image segmentation is necessary for multiple purposes; for example, counting objects, measuring the two-dimensional (or three-dimensional) distribution of the objects, measuring the shape or appearance of individual objects, recognizing the individual objects, localizing objects for tracking, removing unnecessary regions, etc.

It is important to note that image segmentation is the most difficult task among all image processing tasks. Even though human beings perform image segmentation without any difficulties, computers often suffer from its difficulty. In fact, we have not had any perfect segmentation method yet even for human face separation from a picture. Biological images often have far more difficulties than face images. This is because target objects in biological images have ambiguous boundaries and thus are difficult to be separated from the background and other objects. Furthermore, all the difficulties listed in the Introduction (such as low resolution) make segmentation a difficult task.

Table 4 lists typical image segmentation methods, which have been developed for general (i.e. non-biological) images. Those methods are overviewed below, except for binarization, which has already discussed before. Again, there is no perfect segmentation method, especially for biological images. It will be an important future work to develop new methods specialized for biological images.

**Table 4:** A list of image segmentation methods

Name	Methodology	Merit	Demerit
Image binarization	See Table 6	Appropriate when the target object is comprised of only bright pixels (or dark pixels)	Limited applicability (however, note that several binarization methods can be extended for multi-level thresholding. For example, by using two thresholds, an image is partitioned into bright regions, mid regions, and dark regions.)
Background subtraction	Detect target objects by removing the background part	Appropriate when target objects are distributed over the background	The background image is necessary. Especially when the background is not constant, some dynamic background estimation is necessary
Watershed method	Representing an image as a three-dimensional surface, and detecting its ridge lines, i.e. watershed	Even if gray-level change is not abrupt, it is possible to detect its peak as an edge	Appropriate preprocessing is necessary for suppressing noises
Region growing	Iterative. If neighboring regions have similar properties, combine them	Simple	Inaccurate due to its local optimization policy
Clustering	Grouping pixels with similar properties	Simple. Popular clustering algorithms, such as k-means, can be used	Difficulty in balancing locational proximity and pixel value similarity
Active contour model (snakes)	Optimally locating a deformable closed contour around a single target object	Robust by its optimization framework. If the contour of a target object is invisible, it still provides closed contour	Only for a single object. Difficulties of dealing with unsmooth contours. Usually, characteristics of the region enclosed by the contour are not considered
Template matching and recognition based method	Finding pixels or blocks whose appearance or other characteristics are similar to reference patterns of the target object	Capable of stable segmentation by using various pattern recognition theories for evaluating the similarity	Computationally expensive. Often a sufficient number of reference patterns are necessary for realizing stability
Markov random field (MRF)	An integrated method to optimize the segmentation result considering the similarity of neighboring pixels	Accurate and robust. Highly flexible and capable of using various criteria	Computationally expensive. Difficult to implement

### Background subtraction

Background subtraction is a simple method of target object separation from an input image using a background image. Background image is an image capturing no target object. For example, if we have a background image of an empty room, we can detect objects in the room by subtracting the background image from the input image. Note that the background image and the input image should be taken from the same viewpoint. If they were taken from different viewpoints, we need to register them in advance to background image subtraction for compensating the perspective distortion. (Image will be detailed later.)

**Fig. 6:**
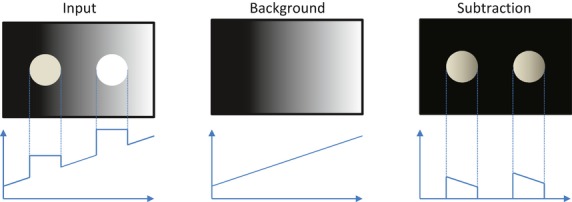
Background subtraction. If there is a background image, it is possible to detect targets in an input image. Note that the pixel intensity value of the detected targets in the subtraction image can be different from its original value. If it is necessary to retrieve the original intensity value, it should refer to the pixel value of the original image at the pixel with a non-zero value in the subtraction image.

Figure 6 illustrates the process of background subtraction. Note that if the pixel value of the background image is brighter than that of the input image at a certain pixel, the subtraction value becomes a negative value. Therefore, we usually need to adjust the pixel value in the subtracted image suitable for visualization. The simplest way is to use the absolute value of the subtraction value. Also note that, as indicated in Figure 6, the pixel intensity value of the detected targets in the subtraction image can be different from its original value. If it is necessary to retrieve the original intensity value, it should refer to the pixel value of the original image at the pixel with a non-zero value in the subtraction image.

The most important procedure for background subtraction is the acquisition of background image *B*(*x*, *y*). If it is possible to exclude all targets from the observation area at a certain time, it is easy to use the image of this time as a background image. If it is impossible, we need to “estimate” the background image in some way. For video sequence *I*_*t*_ (*x*, *y*), *t* = 1, …, *T*, one idea is to use a temporal median filter, which derives the pixel value by a median value over time, that is, *B*(*x*, *y*) = Median_*t*_ *I*_*t*_ (*x*, *y*). This estimation assumes that the probability of a target object appears at (*x*, *y*) is <50% throughout the entire video frames. In other words, the probability that (*x*, *y*) is showing its background value is higher than 50%.

In a more elaborated manner, we can consider a problem of classifying each pixel to the foreground (i.e. lies on a target object) or background. In this case, we need to know the characteristics of foreground and background. For example, if we know the pixel value distributions for foreground and background pixels respectively, we can classify a pixel into foreground or background (See Template matching and recognition based method).

### Watershed method

Watershed is an image segmentation method, which has been used for bioimages, such as cell nuclei segmentation (Coelho *et al*. 2009). The term “watershed” means the ridge lines of a three-dimensional surface like a ground-surface. Roughly speaking, the watershed method considers a region surrounding a closed ridge line as a partitioned region. Therefore, its property depends on how a surface is derived from an input image.

Any gray-scale image itself can be represented as a three-dimensional surface by considering a gray-scale value at a pixel is a height at the pixel location; this representation, however, is not appropriate for the watershed method. This is because its local peaks often do not correspond to a segmentation boundary; consider an image where a white-filled circle lies on a black background. It is clear that any boundary pixel of the circle does not correspond to a local peak of the gray-scale surface.

Instead, a gradient image of the gray-scale image can be used for the watershed method. The pixel value of the gradient image is an absolute gradient value at the pixel. In the above example, only pixels at the boundary of the circle have large gradient values and other pixels have about zero gradient values. Fortunately, the gradient image satisfies the above requirement that its local peaks correspond to the boundary. Therefore, by detecting continuous pixels connecting the local peaks, it is possible to partition an image into regions.

When we use a gradient image, we need to be careful about noise. Gradient is very sensitive to noise because even a small fluctuation in gray-scale level is emphasized in the gradient image. Some preprocessing such as smoothing is necessary.

Another choice of creating three-dimensional surface is to use the distance transform. Given a binary image, the distance transformation counts the distance to the closest black/white boundary at each pixel. Let us consider a binary image with the result of a rough segmentation; the black regions of the binary image are ambiguous regions and segmentation boundary should be located in the black region. In contrast, each white region is considered as a central region of a segment. The distance transformation is then performed on the binary image to count the distance at individual black pixels from their closest white pixels. By considering the distance value as the height of the three-dimensional surface, the watershed of the surface corresponds to equidistant lines from neighboring white regions and thus a segmentation boundary. This is a kind of Voronoi decomposition of a binary image.

### Region growing

Region growing is a classic and simple segmentation method. Its process begins with treating each pixel as a segment. Then, if neighboring segments have any similar characteristics (e.g. color), they are merged to be a new segment. By repeating this merging process until convergence, a segmentation result is obtained. We can use various measures for evaluating the similarity between neighboring segments. Clearly, region growing is weak against noises (in the worst case, some noisy pixels are isolated as small segments). Thus, it will be better to apply a smoothing method prior to region growing.

### Clustering

Generally, clustering (Jain *et al*. 1999) is a method of partitioning a set into subsets according to some criterion. The elements grouped in a subset will have mutual similarity. Clustering can discover latent groups in a large number of samples. For example, we can perform image clustering. If we have many images, image clustering reveals their latent groups.

Clustering can be used for image segmentation. Clustering-based image segmentation begins by representing an *M* × *N* image as a set of *M* × *N* vectors, *P* = {(*x*, *y*, *l*(*x*, *y*))}, where *x* and *y* represent the location of each pixel and *l*(*x*, *y*) represent some feature vector of the pixel. A typical example of *l*(*x*, *y*) is an RGB color vector. By applying some clustering method to the set *P*, the set is partitioned into groups having not only similar locations but also similar feature vectors, that is, an image segmentation result. A key point of this method is to represent each pixel with its location, *x* and *y*. Without the location, pixels having similar features are grouped regardless of their location.

There are many clustering methods. K-means is a classic and still widely-used method. Its algorithm is described as follows:

1. Initialize *K* “centroids”, *r*_*k*_, *k* = 1, …, *K*. One possible way of the initialization is a random selection of *K* vectors from *P*.
2. Repeat the following two steps until convergence.
3. For each vector in *P*, select its closest centroid from *K* centroids. Eventually, we have subset *P*_*k*_ & *P* which comprises vectors whose closest centroid is *r*_*k*_. Note that ∪_*k*_*P*_*k*_ = *P* and *P*_*k*_ ∩ *P*_*k*′_ = ∅ (empty set) if *k* ≠ *k*′.
4. Update *r*_*k*_ as the average of vectors in *P*_*k*_.

**Fig. 7:**
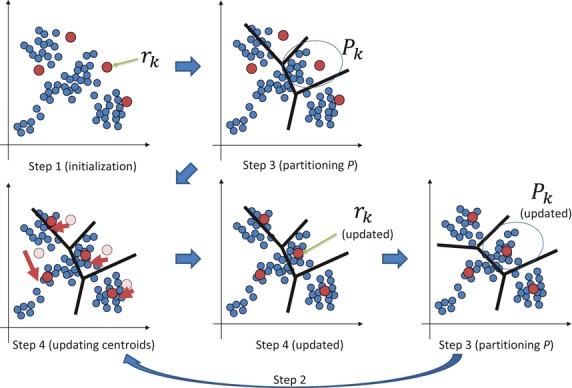
K-means algorithm. The number of centroids is fixed at 4 in the figure.

Figure 7 illustrates the above four steps. Note that this K-means algorithm is applicable not only to the above image segmentation problem but also any clustering problems.

One weak point of K-means is that we have to specify the number of groups, *K*, in advance. In the segmentation task, this means that we have to specify the number of segments and this specification is not trivial. This weak point is relaxed by a trivial modification. If vectors in a subset *P*_*k*_ have a large variance, we partition *P*_*k*_ into new subsets by local clustering only for *P*_*k*_ (this can be called, hierarchical K-means). Another way is to redo K-means just by increasing *K*. In fact, K-means have several variations to overcome weakness. The LBG algorithm and ISODATA are considered as such variations.

### Active contour model

Active contour model, also called SNAKES, is the method to find a closed contour of a target object (Kass *et al*. 1998). Although it is a kind of curve detection method, it also can be considered as a segmentation method. The strength of the active contour model is that it formulates the contour detection problem as an optimization method. In other words, it tries to find out the best contours from all possible contours according to a certain criterion. Consequently, it can provide a reasonable contour even under noises or ambiguous edges.

A typical criterion on optimizing the contour is a sum of edge-ness and smoothness. This means that the active contour model assumes that the contour is smooth and located around some edges. In other words, the (original) active shape model will not be suitable for segmentation of objects having keen corners.

The level set method is an extension of the active contour model, because it can deal with multiple contours. Its main idea is to use a smooth function *f*(*x*, *y*) over an image and consider its cross-section, *C* = {(*x*, *y*) | *f*(*x*, *y*) = 0}. For example, if the function *f* seems like a two-dimensional quadratic function, *C* looks like an ellipsoidal contour. If *f* has two peaks, *C* will comprise two contours. Like the active contour model, the shape of function *f* is determined by considering the smoothness and the edge-ness.

The active shape model is similar to the active contour model, but different at the point that it represent segmentation contours by a combination of a mean (i.e. standard) shape and typical shape variations. Accordingly, its flexibility in forming segmentation contours is less than the active contour model. However, the active shape model is often useful when the target object undergoes some typical deformations rather than arbitrary deformations. The typical deformations are derived by principal component analysis (PCA), which is a popular statistical analysis method to understand typical variations in a dataset.

### Template matching and recognition based method

Formally, image segmentation can be considered as an image pattern recognition problem. This is because, like the binarization problem, image segmentation is a problem of determining a class of each pixel. For example, we can consider classes such as nucleus class, Golgi class, mitochondrion class, etc., for cell image segmentation.

A simple realization of image segmentation by using pattern recognition techniques is template matching. Let us consider the simplest case where a template for the class *c* is defined as a typical pixel value of *c*. After preparing *K* templates, *r*_*c*,1_, …, *r*_*c*,*K*_ for each class *c*, the most similar template is found for each pixel (*x*, *y*) of the input image. A possible way to evaluate the similarity is the distance ∥*l*(*x*, *y*) – *r*_*c*,*k*_∥, where *l*(*x*, *y*) is the pixel value at (*x*, *y*). The class *c* of *r*_*c*,*k*_ giving the minimum distance among all {*r*_*c*,*k*_} is the recognition result at (*x*, *y*). The recognition results on the input image show the image segmentation result.

This simple realization can be extended in various aspects. First, it is possible to use a small region, such as a square block, instead of a pixel as a unit of recognition. By this extension, it is possible to perform segmentation considering specific textures (i.e. spatial patterns). Second, we can use more elaborate similarity evaluation. This means that we can incorporate various pattern recognition techniques for image segmentation. Third, it is possible to consider some class consistency of neighboring pixels. This extension is closely related to Markov random field (MRF) as follows.

### Markov random field

Binarization by optimization on MRF (see Optimization-based binarization) can be extended to image segmentation. While each pixel was assigned to one of two classes (black and white) for binarization, each pixel can be assigned to arbitrary and more than two classes for image segmentation. Like binarization, the class decision depends on two factors. The first factor is class-likelihood, i.e. the similarity to each class. This can be calculated by the above recognition-based method. The second factor is smoothness that neighboring pixels are better to be assigned to the same class is introduced. A difference from binarization is that the optimal segmentation problem (i.e. the optimal class decision problem at individual pixels) is now computationally difficult to solve due to the increase of classes. Graph-cut is still applicable to the problem but several modifications (such as “alpha-expansion”) are necessary. Other optimization algorithms, such as Markov chain Monte Carlo (MCMC), have also been used. Many research activities on this topic indicate that there is still no perfect image segmentation method on MRF.

## Visual object tracking

Visual object tracking is an important image processing technique for biological videos. In the image processing research community, many object tracking methods have been proposed so far (Yilmaz *et al*. 2006). This fact indicates that visual object tracking is still an open problem even for daily-life images. As noted before, bioimages have more difficulties than daily-life images and thus object tracking in bioimages is very challenging. For example, the task of tracking GFP protein molecules will suffer from multiple targets with almost identical appearances moving around a noisy background. Consequently, visual object tracking for bioimages is also an open problem and needs to be improved by further efforts.

**Fig. 8:**
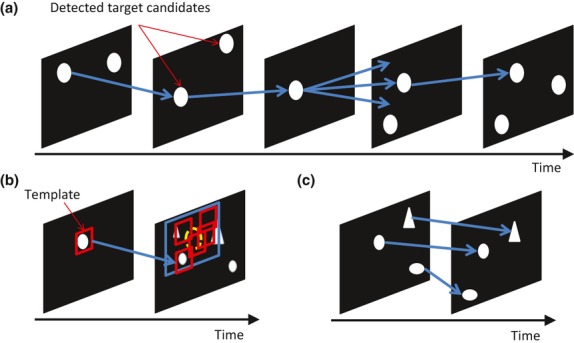
Visual object tracking. (a) Connecting the detected target locations. In some frames, multiple candidates exist and thus we need to select the best candidate for having, for example, a smooth trajectory. (b) Template-based tracking. Around the previous target location, the most similar (or the least dissimilar) location is selected as the current target location. (c) Multiple object tracking. The optimal one-to-one correspondence should be determined between each pair of consecutive frames.

Figure 8a illustrates the visual object tracking problem. Generally, two sub-problems are necessary for solving the problem. The first sub-problem is to “detect” one or more target objects at each frame. This is necessary because the location of the target is usually unknown. This problem can be solvable image segmentation and/or pattern recognition. The second sub-problem is to “connect” the detection results over all frames to form one or more trajectories. By solving the connection sub-problem, we can understand the speed and the direction of each target.

If there is only a single object detected at each frame, it is easy to form a trajectory just by connecting the sequence of the detection results over the frames. In more general cases, however, this sub-problem is not trivial. If we have *K* detected objects at every frame, we have to establish a one-to-one correspondence between two consecutive frames, as shown in Figure 8c. This is called a matching problem and we need to find the best correspondence among *O*(*K*!) possible correspondences. In more difficult but probable cases, there are some spurious detected objects and misdetection results, in addition to newly appearing targets, disappearing targets, and overlapping targets.

Note that these two sub-problems are sometimes solved in a two-step manner (that is, detection and then connection) and sometimes solved in a one-step manner (that is, detection and connection is done simultaneously). In the latter case, two sub-problems are mutually dependent. For example, a target may be detected at a location where the connected trajectory becomes smoother. This is useful when it is difficult to expect accurate target detection results only by solving the detection sub-problem independently.

Table 5 shows four major properties for classifying object tracking methods. Among them, the last property, the optimization strategy, will need a further explanation. Generally, the visual object tracking problem can be formulated as an optimization problem where (i) the detected target locations at individual frames and (ii) the connection between the detected targets are to be optimized. As a criterion for the optimization, some goodness, such as a smoothness of the trajectory and a probability (called likelihood) of a target existing at a certain location, is evaluated.

**Table 5:** Four major properties on classifying visual object tracking methods

The number of targets	*Single* Only one target exists on each frame	*Multiple* Many targets exist on each frame. Often, the number of targets changes over time
Target shape	*Point* During the tracking process, each target is treated as a point	*Arbitrary shape* Each target is a (often deformable) object represented as a region or contour
The order of target detection and tracking	*One-step* During the tracking process, target location and smooth trajectory are determined simultaneously	*Two-step* Candidates of the target location are first detected at each frame and then selected to form a smooth trajectory
Trajectory optimization strategy	*Online* Every time a frame comes, the tracking result to the frame is determined. Suitable for real-time tracking	*Offline* After all frames are obtained, the tracking result is determined. Thus, the tracking result at frame *t* can be determined fully using any other frames (even a future frame *t*′ *> t*). Unsuitable for real-time tracking, but robust

For solving this optimization problem, there are two choices – online optimization and offline optimization. Online optimization, which is more widely used for the solution of object tracking problems, determines the detection and connection result till the *t*th frame when the *t*th frame is inputted. Identically, online optimization determines the detection result at the *t*th frame and the connection result between *t*−1 and *t* at *t*. This result will not be changed at later frames. Online optimization is suitable for real-time processing. It is, however, weak against failures at beginning frames; if it fails at *t*, it is difficult to recover at later frames. In the worst case, the failure affects the later results more severely. In contrast, offline optimization starts its optimization after all the frames are inputted. Therefore, even if it is difficult to determine the tracking result at *t* by itself, the later frames will help the determination. This is helpful for the object tracking on biological videos where it is difficult to detect the same target with no error at all the frames.

### Tracking by template matching

The simplest tracking method is the template matching-based method, which is illustrated in Figure 8b. In this method, we need to prepare an image of the target object as a template image. Then, the location with the highest similarity to the template on each frame is considered as a new target location. The new target location at *t* is often searched around the previous location at *t*−1 for forming a smooth temporal trajectory.

The idea of this simple tracking method can be extended in various directions. For example, (i) template can be fixed or updated in a frame-by-frame manner. (ii) We can use various criteria for evaluating the similarity (or dissimilarity). (iii) We can use multiple templates for dealing with change of target appearance.

Introducing image recognition techniques to the template matching-based tracking is a promising approach. Instead of using a simple similarity, we can use any image recognition technique to evaluate goodness (i.e. likelihood probability) of a certain position as a target location. Recently, to fully use the power of image recognition, discriminative tracking methods have been developed, where some “badness” of a certain position is also introduced in addition to some goodness. This “badness” evaluates, for example, how a certain position looks like background. Consequently, a large “badness” at the position avoids that a tracking result pass through the Support Vector Tracker (Avidan 2004) is a good example.

### Other tracking methods

Table 6 shows major tracking methods used in ordinary (i.e. non-biological) video images and Table 7 shows their properties (according to the four properties in Table 5). Note that most tracking methods are originally developed for single-object tracking, but extendable for multiple-object tracking. For example, in Smal *et al*. 2008; a particle filter tracker is used for multiple object tracking, although conventional particle filter trackers have often been developed for single object tracking. Recently, a promising offline tracking method based on integer linear programming (Jaqaman *et al*. 2008; Berclaz *et al*. 2011; Bise *et al*. 2011) has been proposed. This method can provide a globally optimal tracking result for multiple objects while avoiding exponential (i.e. intractable) computations.

**Table 6:** Major visual object tracking methods

Tracking method	Methodology	Merit	Demerit
Template matching	Search the similar pattern to a template image of the target object around the position estimated at the previous frame	Simple. Extendable	Weak to deformations and occlusion
Lucas- Kanade tracker	Similar to the template matching method but more efficient by using a gradient-based solution	More efficient than template-matching. Capable of dealing with rotation and other parametric deformations, such as affine	Difficult to deal with a large displacement and deformation between consecutive frames
Mean-shift	Represent the target object by color histogram	Robust to deformation. An efficient search by combining gradient-based strategy	Weak to occlusion and existence of similar objects
Kalman filter	Determine the current target position by integrating the current frame and a prediction result	Robust if the assumptions hold true. Efficient by linear computation	Several hard assumptions in target motion
Particle filter	Estimate the distribution of the target position with multiple hypotheses generated and evaluated by some fitness	Robust by its multiple (i.e. parallel) search nature and statistical validity	Large computations. Ambiguity on determining the position from the estimated distribution
Dynamic programming-based tracking	Solve the tracking problem as a globally optimal path problem in spatio-temporal space	Even if severe distortions, such as occlusion, happen in several frames, we can expect a stable result	Unsuitable for real-time application. Huge computations
Integer linear programming-based tracking	Similar to dynamic programming-based tracking, but more efficient by introducing specific constraints	The same as above	The same as above, but less computations

**Table 7:** A classification of visual object tracking methods

Tracking method	The number of targets	Target shape	The order of target detection and tracking	Trajectory optimization strategy
Template matching	Single/Multiple	Arbitrary	One-step	Online
Lucas–Kanade tracker	Single/Multiple	Arbitrary	One-step	Online
Mean-shift	Single/Multiple	Arbitrary	One-step	Online
Kalman filter	Single/Multiple	Arbitrary	One-step	Online
Particle filter	Single/Multiple	Arbitrary	One-step	Online
Dynamic programming-based tracking	Single/Multiple	Arbitrary/Point	One-step/two-step	Offline
Integer linear programming-based tracking	Multiple	Arbitrary/Point	Two-step	Offline

Active contour models and the level set method can be used for tracking deformable objects, such as motile cells. This tracking method can be realized just by optimizing the contour around the target object. One important clue for robust tracking is the fact that the object shapes at consecutive frames are similar (when the frame rate is high enough); consequently, the location and shape of the optimized contour at a frame can be a good initial value for the next frame. For tracking a deformable object, which will divide into two or more pieces (such as cell mitosis), the level set method can be applicable (Yang *et al*. 2005).

### Comparison of trajectories

The result of object tracking, i.e. the temporal trajectory of the target, is often used for plotting a velocity histogram and a moving direction histogram. It is also useful to analyze some other characteristics, such as a deviation from a normal temporal trajectory. In the latter case, we need to compare the trajectory showing the current tracking result and some reference trajectory.

**Fig. 9:**
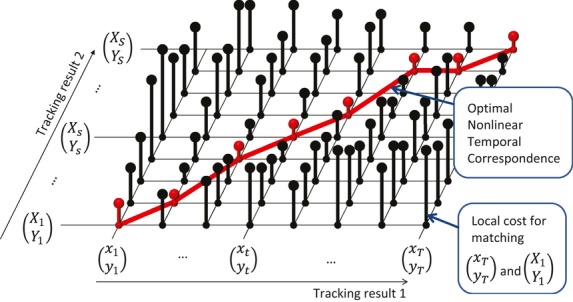
Dynamic time warping (DTW) for comparing two tracking results, that is, two temporal trajectories. Using dynamic programming, the optimal correspondence, which is represented as a path (1,1), …, (*t*,*s*), …, (*T*,*S*) having smaller local costs, is derived. Note that the lengths of the trajectories (*T* and *S*) can be different.

Dynamic time warping (DTW), or DP matching, is a technique to compare two temporal patterns and has been used in speech recognition and character recognition from the 1970s. Figure 9 illustrates DTW for a pair of tracking results, i.e. temporal patterns. One main function of DTW is to provide the optimal nonlinear temporal correspondence between two temporal patterns. The nonlinearity is useful for dealing with nonlinear temporal fluctuation. Another function is to evaluate the similarity or dissimilarity between two temporal patterns. Different from Euclidean distance, DTW can provide a similarity even when two patterns have different lengths.

### Optical flow

**Fig. 10:**
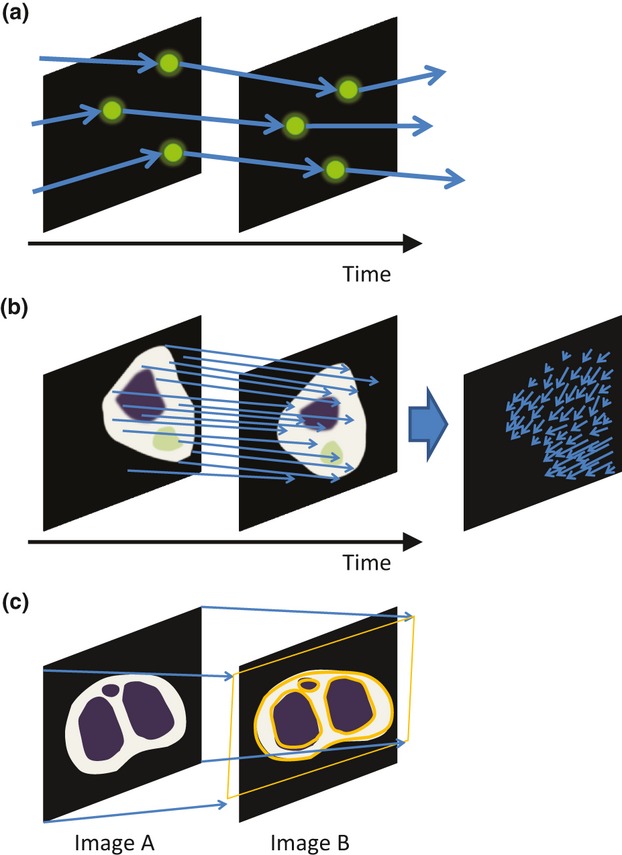
Difference among three similar image processing techniques. Visual object tracking (a) tracks one or multiple objects over all video frames (more than two frames). Usually, it is assumed that each individual object will have the same motion and thus a tracking result will show a sparse motion field. Optical flow (b) provides motion at every pixel by observing a pair of consecutive frames (not all the frames). Accordingly, it provides a more “dense” motion field and an object will have different motions at different positions inside the object. Image registration (c) matches a pair of images using a 2D-2D mapping function (this figure uses an affine transformation function for registration). The images need not be a pair of consecutive frames in a video; thus, we can apply image registration between, for example, face images of different persons.

Optical flow (Beauchemin & Barron 1995) is a technique to estimate the motion at all the pixels at each frame. As shown in Figure 10a,b, optical flow can provide a pixel-wise “dense” motion field on the entire image than visual object tracking. Thus, optical flow is useful to analyze not only the motion but also the deformation of flexible objects. It is possible to consider that optical flow is a special case of multiple point object tracking; specifically, we can consider every pixel at the *t*th frame is an individual point object and estimate its displacement at the next (*t* + 1)th frame.

Optical flow, however, is often tackled with different methodologies from visual object tracking. This is because each pixel has less information than a larger object (represented by a template) and its motion is more ambiguous. As an extreme example, assume a pair of consecutive frames just showing totally black images. In this example, we can define arbitrary motion for each pixel. Even in more probable cases, the tracking result just by single pixel information is very fragile to noise. For example, assume that the image of the (*t* + 1)th frame is a bit brighter version of the *t*th frame. In this example, the correct optical flow may be “no flow,” although a pixel-wise tracking result may show some motion.

One may consider that it will be better to use a block (a small square region) around each pixel instead of a single pixel and then, like the template matching-based tracker, determine the motion of the block. This simple remedy works better and is sufficient for some applications (in fact, MPEG uses this motion estimation method). However, it is not sufficient for many cases since the motion of individual pixels (i.e. blocks) are estimated independently.

Consequently, for solving the optical flow problem, some dependency between the motions of neighboring pixels is assumed. The typical dependency is smoothness that the motion of a pixel is similar to the motions of its neighboring pixels. By introducing a dependency, the optical flow problem is now formulated on a Markov random field, like the image binarization problem and the image segmentation problem, and the motions of individual pixels are determined simultaneously by some optimization strategy. It should be noted that this is a two-dimensional optimization problem like the image segmentation problem and thus we have to consider how we can derive an efficient solution while avoiding intractable computations.

A typical method to solve the optical flow problem is an iterative method called the Horn-Schunck method, where the smoothness is considered along with a fundamental equation for motion estimation. The equation is called the optical flow constraint equation and is defined as:


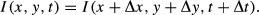


This equation assumes that for each pixel (*x*, *y*) of the *t*th frame, there is a corresponding pixel with the same gray-scale value at (*x* + Δ*x*, *y* + Δ*y*) of the (*t* + 1)th frame. Note that the time interval between the frames *t* and *t* + 1 is Δ*t*. The Horn-Schunck method, therefore, still has a weakness to changes in the gray-scale value but often shows reasonable performance (by the effect of smoothness).

## Image registration

Image registration is the technique to fit an image to another image. Figure 10c illustrates image registration. Usually, those images subjected to image registration are different but similar to each other. In other words, image registration is to fit a reference image to its deformed version or *vice versa*. The purpose of image registration is to overlay two images while fitting them to each other flexibly, that is, elastically. If there is any difference even after the fitting, those images have some intrinsic difference. Another purpose of image registration is to understand relative deformation of an image to the other image. The optimized fitting function between two images directly represents the deformation.

Image registration is useful for various bioimage analysis tasks. Generally, comparison between two biological objects is always a promising task of image registration. One promising task is to understand the shape change and/or appearance change of an embryo by its development and growth. Another promising task is anomaly analysis of a cell or a single organelle using its relative deformation to a healthy one or wild type. Image registration is also applicable to various phenotype analysis tasks because they deal with some analysis in shape and appearance.

**Fig. 11:**
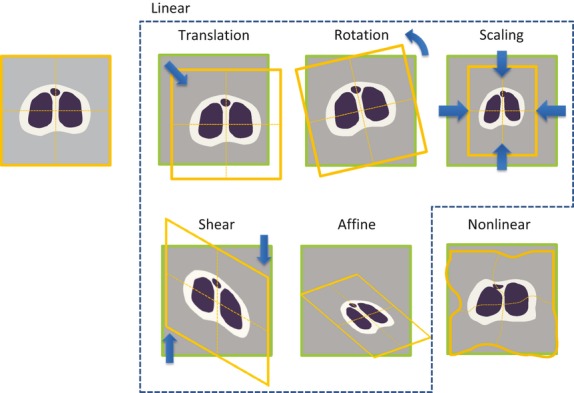
Geometric transformation functions for image registration. Note that affine transformation is a combination of translation, rotation, scaling, and shear. Also note that linear transformation functions map any straight line to a straight line, whereas nonlinear transformation function maps a straight line to a curve.

Mathematically, the fitting function for image registration is represented as a mapping function from 2D (the x–y coordinate of image A) to 2D (the x–y coordinate of image B) and is called geometric transformation or image warping (Wolberg 1990). The image registration methods are classified by their types of transformation function. This is because the type specifies the range of deformations compensable by the image registration method. If the transformation function is a linear 2D-2D function, it is possible to realize a less flexible image registration method. If the transformation function is a nonlinear 2D-2D function, we will have a more flexible image registration method, which can deal with a wide range of deformations. Figure 11 illustrates linear and nonlinear geometric transformation functions, which are detailed below.

### Image registration by linear geometric transformation

The simplest linear transformation function is a horizontal and/or vertical shift. This shifting transformation is called translation. Rotation and scaling (i.e. zoom-up/down) are also typical linear geometric transformations for image registration. Amore general geometric transformation is affine transformation and perspective transformation. Affine transformation includes translation, rotation, scaling, shear, and their arbitrary combinations. Affine transformation has a property in which parallel lines are still parallel after transformation.

Any linear transformation function is represented as a matrix, that is,


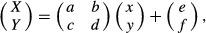


or equivalently,


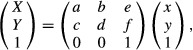


where (*x*, *y*) is the *x–y* coordinate of image A and (*X*, *Y*) is of image B. Parameters *e* and *f* represent translations in *x* and *y* directions, respectively. For rotation, *a* = *d* = cosθ and −*b* = *c* = sinθ. For affine transformation, all six parameters are arbitrary (this explains that affine transformation includes translation, etc., as noted above.) Note that any linear transformation function maps any straight line as a straight line as shown in Figure 11.

The problem of image registration with linear transformation function is to estimate the parameters of the linear transformation. The estimation criterion is typically described as follows:


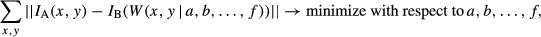


where *W*(*x*, *y* | *a*, *b*, …,*f*) denotes the above geometric transformation function, or the warping function, and maps (*x*, *y*) to (*X*, *Y*) according to the six parameters *a*, *b*, …, *f*. If we only assume translation, we have to estimate two parameters *e* and *f* while fixing *a*, *b*, *c*, *d* at 1, 0, 1, 0, respectively.

It should be emphasized that even though the geometric transformation function is rather simple, its estimation problem is not trivial. In fact, there is neither a direct nor analytical solution to estimate the optimal parameters. This is because *a*, *b*, …, *f* are the parameters inside the nonlinear function, *I*_B_. The most naïve alternative is to try all possible parameter values to find the best one. This is possible if we assume only translation within a small range. If the translation range becomes wider, it is possible to use the Fourier transformation for an efficient solution (Zitová & Flusser 2003). If we assume affine, it is practically impossible and thus we need to use a more elaborate method.

**Fig. 12:**
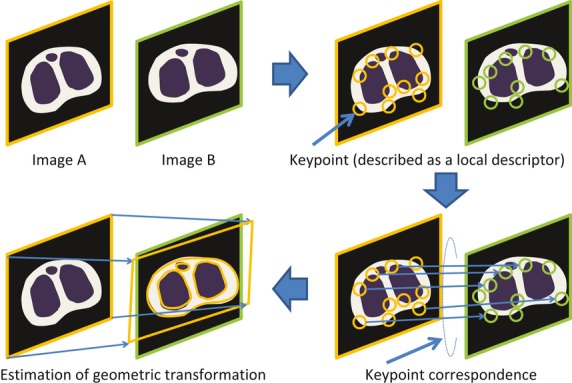
Estimation of the geometric transformation function by keypoint correspondence. First, keypoints are detected at each image and then each keypoint is described as a feature vector, called local descriptor. Second, keypoint correspondence is established by using similarity between local descriptors. Finally, the geometric transformation function is estimated using the keypoint correspondence.

Recently, the estimation methods based on keypoint correspondence become more popular. As shown in Figure 12, the methods are comprised of three steps.

1. Detect and describe keypoints.
2. Establish keypoint correspondence between images.
3. Determine the geometric transformation, using the correspondence.

A keypoint is defined as a pixel at a distinctive location. For example, a corner pixel of an edge line is detected as a keypoint. Each keypoint is then described by a vector. Specifically, a small region is specified around each keypoint and a set of feature values are calculated from the region. An example of the feature value is the gradient value in a specific direction. The set of feature values are represented as a vector, called a local feature vector or a local descriptor.

Detection and description of keypoints are very important for better correspondence, and thus for better image registration. Detected keypoints and their local descriptions should be invariant to geometric transformations. This means that the same keypoints having the same local descriptors will be detected at an image and its deformed version. Consequently, we can find the correct correspondence between keypoints by evaluating the similarity between their local descriptors — if they have a larger similarity (or, equivalently, a smaller Euclidean distance), they will correspond to each other. Mikolajczyk & Schmid (2005) have provided a comparative study on this matter. In their paper, DoG, Harris-Affine, and Hessian-Affine are used as detectors, and SIFT (Scale-Invariant Feature Transform), SURF (Speeded Up Robust Features) (Bay *et al*. 2006) and their extensions, shape and context, are used as descriptors.

The established keypoint correspondence is useful to estimate the geometric transformation function, *W*(*x*, *y* | *a*, *b*, …, *f*). This estimation is far easier than the previous estimation scheme because we no longer need to consider the images *I*_A_ and *I*_B_. Specifically, the problem becomes


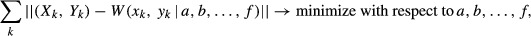


where (*x*_*k*_, *y*_*k*_) and (*X*_*k*_, *Y*_*k*_) are the *x–y* coordinates of *k*th corresponding keypoints. Since we now consider six parameters, we can determine them by three keypoint correspondences. If we have more keypoint correspondences, the above minimization problem is a least square problem and thus still solvable analytically. Note that we can extend this methodology to perspective transformation.

Unfortunately, the least square solution is usually not satisfactory because the keypoint correspondence established by the similarity of local descriptors sometimes make a big mistake due to the existence of unexpectedly similar local regions. Consequently, RANSAC (Fischler & Bolles 1981) is often used in the keypoint correspondence-based methods. RASAC is a robust estimation method; first, three (or more) correspondences are randomly selected among *K* correspondences and the parameters are estimated using the correspondences. Then, it is evaluated how the remaining correspondences agree with the geometric transformation functions determined by the estimated parameters. If the current parameters can have enough agreements, the parameters are selected as the final answer. Since RANSAC does not use all the correspondences for deriving parameters, it is robust to erroneous correspondences.

### Image registration by nonlinear geometric transformation

Nonlinear image registration, or deformable template, realizes more flexible image registration than linear image registration. Different from linear image registration, nonlinear image registration can map a straight line as a curve as shown in Figure 11. Mathematically, nonlinear image registration is equivalent to optical flow because optical flow is also represented as an arbitrary 2D-2D function. As shown in Figure 10b,c, optical flow is usually applied to a pair of consecutive video frames and image registration is applied to an arbitrary pair of images. This difference, however, is rather superficial – in fact, any optical flow method can be applicable to image registration tasks and *vice versa*.

There has been vast research on nonlinear image registration (Glasbey & Mardia 1998; Jain *et al*. 1998; Redert *et al*. 1999; Uchida & Sakoe 2005). Like optical flow, some dependency between the mapping of (*x*, *y*) and that of its neighborhood is often introduced. Thus, the problem becomes a kind of MRF.

## Image pattern recognition

### General idea of image pattern recognition

Image pattern recognition is a task to assign a predefined class label to an image (or a part of an image). As noted before, OCR is a typical image pattern recognition task, where an input image is assigned to one of character classes. Diagnosis of an embryo, or a single cell, or a subcellular organelle using its imaging result is also an image pattern recognition problem. For the simplest diagnosis, it is reduced to a two-class recognition problem, that is, normal or abnormal. As also noted before, image segmentation is also related to image pattern recognition. In fact, interpretation of each segment is an image pattern recognition task.

Image pattern recognition is comprised of two modules: feature extraction and classification. Feature extraction is the module to convert an input image as a set of values, that is, a vector. In the simplest case, the input image (say, an *N* × *N* gray-scale image) is represented as a set of *N*^2^ gray-scale values, that is, an *N*^2^-dimensional vector. It is possible to use other representations. A histogram of the gray-scale values is a popular choice. Use of statistics, such as the mean and the variance of gray-scale values, is also possible. Other possible feature representations will be described later.

Classification is the module to classify the input feature vector into a class according to some rule, called a classifier. A classifier is trained automatically using patterns whose class is known. This training mechanism of the classifier is called machine learning and feature vectors for training are called training patterns. The class label attached to each training pattern is called the ground-truth. We need to train a classifier to classify the training patterns correctly. The patterns subjected to the trained classifier are called test patterns. The test patterns are assumed to be “unseen” patterns and thus we do not know their correct label. However, for evaluating the performance of the trained classifier, we often use the test patterns with ground-truth. Usually, the test pattern set and the training pattern set should be independent.

As an example, consider a problem of classifying a person into two classes, “healthy” and “unhealthy” by using her/his height and weight (this example is, therefore, not an “image” pattern recognition problem; however, the nature of this classification problem is exactly the same for image pattern recognition). In this case, the input feature vector is a two-dimensional vector (height *h*, weight *w*).

**Fig. 13:**
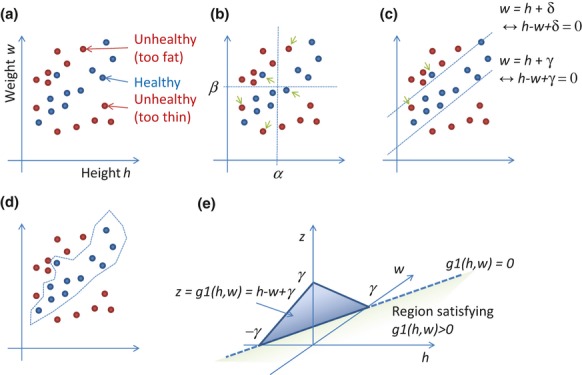
Classification for pattern recognition. (a) Patterns to be classified to two classes, healthy and unhealthy. (b) Classification by using two features independently. The patterns with a green arrow are to be misclassified. (c) Linear classification. The classification boundary is plotted by dash lines. In this case, the classification is done considering the dependency between height and weight. (d) Nonlinear classification. Classification boundary can be an arbitrary curve. (e) A linear discriminant function for the linear classification of (c).

Figure 13a illustrates training patterns distributed on a two-dimensional space. This space is called a feature space. In the feature space, each pattern is represented as a point. The dimensionality of the feature space is the same as the dimensionality of the feature vector. If we represent each *N × N* gray-scale image as a *N*^2^-dimensional vector, the feature space becomes an *N*^2^-dimensional space. Unlike the two-dimensional case, it is impossible to observe a pattern distribution in such a high-dimensional space directly. Research in data visualization will help to convert the distribution to be shown in a lower dimensional space.

We generally expect that similar patterns (that is, patterns from the same class) will have similar feature vectors. Consequently, patterns from a certain class often form one “cluster” in the feature space like “healthy” patterns in Figure 13a. It is also possible that patterns from a class form multiple clusters like “unhealthy” patterns. This may happen when the class can be decomposed into subclasses, such as “too thin” and “too fat.”

Figure 13b is a classifier for the training patterns. The classification rule specified by this classifier is: if (*h < α* and *w < β*) or (*h > α* and *w > β*), the person is healthy; otherwise, unhealthy. This classifier is decomposed into two rules as shown in two boundary lines in Figure 13b, that is, “*h < α* or not,” “*w < β* or not.” Each of those rules only uses a single feature and thus cannot deal with the mutual relationship between *h* and *w* directly.

Figure 13c is a linear classifier whose classification rule is: if (*w < h + δ* and *w > h + γ*), the person is healthy. This classifier is called a linear classifier because its rule (e.g. *w < h + δ*) is a linear in equation of *h* and *w* and, equivalently, its class boundary becomes a line. This classifier is also decomposed into two rules and each of them uses both *h* and *w* for reflecting their relationship so that if *h* is large, *w* also tends to be large.

Figure 13d illustrates a more flexible classifier that can classify all the training patterns. It uses a flexible curve instead of lines for realizing this “perfect” class boundary. It is worth noting that this perfect classifier often shows poorer performance than the linear classifier of Figure 13c. This happens due to outliers in the training patterns. The outliers are patterns that deviate from the main cluster(s) of the class. Since the flexible curve makes it easy to separate all the training patterns including outliers, the resulting over-fitted boundary may fail to classify unseen test patterns. In contrast, the linear classifier cannot over-fit to the outliers and thus is less sensitive to them.

### Feature extraction

Feature extraction is an important module for pattern recognition. This is because it affects recognition performance drastically. In fact, if we extract a good feature for a recognition task, we can expect good recognition accuracy even with a very simple classification module. In a famous textbook (Horn 1986), its author said “*When you have difficulty in classification, do not look for ever more esoteric mathematical tricks; instead, find better features!*”

So, what is the good feature for image pattern recognition? There are two requirements for the feature. The first requirement is that it is sensitive to the difference of appearance (including shapes, colors, grayscales, textures, etc.) among classes. For example, a feature for OCR should be sensitive to the slight shape difference between “1” and “7”. The second requirement is that it is insensitive to the difference of appearance within a class. For example, a feature for OCR should be insensitive to shape variations of “1” even though they are rather significant.

Unfortunately, these two requirements contradict each other. The first one says “be sensitive to difference in appearance” and the second “be insensitive.” This fact is the nature of difficulties in feature extraction. Many researchers have tried to find better features over the years. Even only for OCR, hundreds of features have been examined (Trier *et al*. 1996) and the exploration toward better features is still going on!

Due to this reason, it is difficult to make a review of features which fits general bioimage recognition. An appropriate feature should be designed for each individual recognition task by carefully observing the appearance, variation, and deformation of target images and how they differ among classes, such as normal and abnormal. Table 8 lists typical features for image pattern recognition. From this table, it is also possible to notice that feature extraction is not a trivial task. For example, linear projection-based feature extraction needs a training step to derive a low-dimensional subspace (which approximates the distribution of target image patterns). Extraction of motion and deformation features requires an optical flow technique and an image registration technique, respectively.

**Table 8:** A list of typical features for image pattern recognition

How feature is extracted?	Where feature is extracted from?	Feature name	Notes
Intuition and/or heuristics	Individual pixel value	Gray-scale feature	Use the input image (bitmap) directly as a feature vector of pixel values
		Color feature	
	Connected component (CC)	Topological feature	After thinning a CC, count its holes, crossing points, etc.
		Moment feature	1st order moment is the center of gravity of the CC
	Line segment, curve, contour	Direction, length, curvature, position	
	Texture	Co-occurrence matrix	Co-occurrence of pixel values at distant pixels
	Texture in local region in the image	Local descriptor based on local co-occurrences (e.g. BRIEF and BRISK)	
Gradient analysis	Entire image (bitmap)	Gradient image/edge image	
	Local region in the image	Local descriptor based on local gradients (e.g. SIFT and SURF)	SIFT and SURF are features invariant to size change and rotation
Frequency analysis	Texture	2D Fourier spectrum, discrete cosine transform (DCT), Wavelet transform, Haar-like feature, linear filters	Linear filters can be interpreted as frequency analysis because they also can be interpreted as a filter in the frequency domain
	Contour	Fourier descriptor	A parametric representation of a contour
Histogram analysis	Individual pixel values	Gray-scale/color histogram	Robust to deformation
	Line segments	Direction histogram	
	Connected components	Size histogram of CCs	Related to pattern spectrum
	Local descriptors in the entire image	Bag-of-features	Using representative local descriptors called visual words, count how many local descriptors are classified to each visual word
Linear projection to a trained low-dimensional subspace	Entire image (bitmap)	Discriminative feature	Obtained by linear discriminant analysis
		PCA coefficient feature	Obtained by principal component analysis (PCA)
Structural representation	Local features or local regions scattered over the entire image	Attributed relational graph, constellation model	Feature represented as a graph whose node is a local feature and edge indicates a geometric proximity of the connected nodes. Graph matching techniques are used in classification module for comparing two graphs
Comparison with another image	Motion field by optical flow	Motion feature (motion vector)	Feature showing the geometric change between consecutive frames. The set of arrows in Figure 10b is a motion feature
	Deformation given by comparison to a reference image	Deformation feature (deformation vector)	Linear or nonlinear image registration technique is used for extraction

**Fig. 14:**
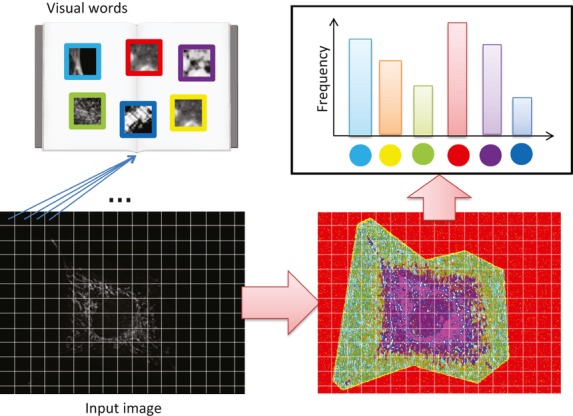
A realization of bag-of-features. An input image is decomposed into parts in some way and each part is assigned to one of the pre-defined *K* visual words. Then, a histogram is created by counting how many parts are recognized as each visual word. This histogram is treated as a *K*-dimensional feature vector and to be recognized by a classifier.

Among feature extraction methods in Table 8, it will be worth detailing bag-of-features since this rather new feature is nowadays widely used for very difficult recognition tasks such as general object recognition. Figure 14 illustrates bag-of-features. For representing an entire image as a bag-of-features, the image is first decomposed into local parts in some way (the same technique was used for keypoint detection and description for image registration). Then, each local part is recognized to one of several pre-defined *K* visual words, which are representative local parts and determined by using local parts from training patterns. Finally, a histogram is created by counting how many parts are recognized as each visual word. This histogram is a bag-of-features and is treated as a *K*-dimensional feature vector and to be recognized by a classifier.

**Fig. 15:**
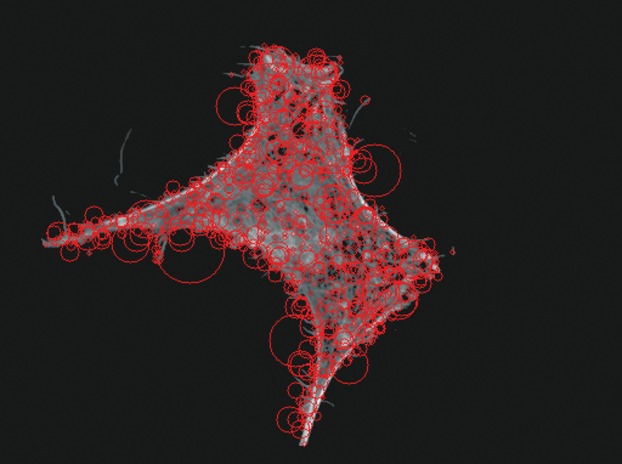
Local regions detected by SURF (Speeded Up Robust Features), which is a method to detect keypoints and describes a small region around each keypoint by a gradient feature. In this figure, each red circle corresponds to a local region detected by SURF. Since SURF has a function to set the size of the local region automatically (according to a condition), the size of the circle varies.

Although an image is decomposed into blocks regularly in Figure 14, it is possible to use local parts scattered over the image. In this case, interesting points (called keypoints) are first detected (often around corner-like points in the image) and a small part around each keypoint is described as a feature vector. Figure 15 shows a result of SURF (Bay *et al*. 2006), which is a method to detect and describe local parts.

### Classification methods

Table 9 lists typical pattern classification methods. There are two main types in those methods: nearest neighbor method and discriminant function method. The nearest neighbor method is the simplest realization of pattern classification. Its principle is to find the training pattern with the minimum difference (or the maximum similarity) to the input pattern among the training patterns (with ground-truth). The class of the selected training pattern is considered as the recognition result. Since the training pattern set is used as a known dictionary in the nearest neighbor method, each training pattern is often called a reference pattern.

**Table 9:** A list of pattern classification methods, where *x* denotes a feature vector of input image and *c* denotes a class

Classification method		How to classify	How to train	Note
Nearest neighbor classifiers	1-nearest neighbor classifier (1-NN classifier)	Select the class of the reference pattern closest to *x*	Just prepare patterns with ground-truth as reference patterns. Thus, no explicit training step. If we need to reduce the reference patterns, some pre-selection might be done in advance	Simple but powerful. Generally, accuracy increases with the number of reference patterns. Many variations by the metric to evaluate “closeness.” Computationally expensive with huge reference patterns
	k-nearest neighbor classifier (k-NN classifier)	Select the majority class of the *k* closest reference patterns to *x*		An improved version of 1-NN. More robust to outliers than 1-NN. Usually, an odd number, say, 3 or 5, is used as *k* to avoid tiebreak
Discriminant function methods	Bayesian classifier	The optimal classifier which uses a posterior probability distribution as the discriminant function	Estimate statistical properties, such as likelihood *p*(*x* | *c*) and the prior probability *P*(*c*), for all classes *c*	Theoretically optimal (by minimizing the Bayes risk) but practically it is difficult to realize because the accurate statistical properties are difficult to estimate
	Linear classifier	Use a linear function of *x* for each class (see Figure 13e) and select the class giving the maximum function value	Error-correcting learning and Widrow-Hoff learning are classic. Recently, SVM is more common	Class boundary is given as a set of hyper-plane in feature space. A special case of Bayesian classification. If each class only has a single reference pattern, 1-NN classifier is reduced to this
	Piecewise linear classifier	Use multiple linear discriminant functions for each class	Consider each cluster of a class as a subclass and train linear classifiers to discriminate subclasses	Class boundary is given as a set of polygonal chains. 1-NN classifier is a special case of this classifier
	Quadratic classifier	Use a quadratic function of *x* for each class	Estimate likelihood *p*(*x*|*c*) as a Gaussian function. Then its logarithm is a quadratic function of *x*. SVM with the second-order polynomial kernel is another choice	Class boundary is given as a set of quadratic curves. A special case of Bayesian classification. Mahalanobis distance is its simplified version
	Support vector machine (SVM)	Determine the class boundary at the center of gap between two classes. By using a so-called kernel, it is possible to have various types of class boundary	Solve a quadratic optimization problem. The problem is to derive the optimally centered discrimination boundary	SVM is a general method to train various discriminant functions in an optimization framework and can provide a linear or a quadratic or a more flexible class boundary. Only two-class classification
	Multilayer perceptron (neural network)	It combines feature extraction and classification modules into one framework. The classification is done by aggregating the outputs from trainable units, called perceptron	Back-propagation is a popular choice. Note that it can train not only classifier but also feature extraction	Huge variations by its inner structure. Perceptron in the simplest case is a linear function whose coefficients are trainable. Nonlinear perceptron is also used
	Voting	Select the majority class in the results by multiple classifiers	If individual classifiers are trained, no further training is necessary	Any classifier can be used. Various voting schemes can be used
Classifier ensemble methods	Boosting	Select the class that wins a weighted voting by multiple classifiers	Classifiers are trained complementary; difficult patterns by a classifier are treated as important patterns on training another classifier. The weight for the voting is a reliability of the classifier	Many versions. AdaBoost is the most popular one. Any two-class classifier can be used
	Decision tree/random forest	Decision tree makes a final classification by hierarchical classifiers. Random forest is a set of decision trees	For a decision tree, ID3 and C4.5 are classic training methods. The key idea of the training is to evaluate the importance of each feature	Random forest is a doubly ensemble method because it is an ensemble of decision trees and each decision tree is also a hierarchical ensemble classifier

The performance of nearest neighbor methods generally improves according to the number of reference patterns. Nowadays, it is possible to store a huge number of reference patterns and also possible to attach the ground-truth to each reference pattern by crowdsourcing (such as Amazon MechanicalTurk), the simple nearest neighbor methods are revived in pattern recognition research. For example, Torralba *et al*. (2008) used nearest neighbor methods on 80 million image patterns.

The discriminant function method is to design a discriminant function *f*_*c*_(*x*) for each class *c*, where *x* is the feature vector of a test pattern. The discriminant function *f*_*c*_(*x*) generally provides a likelihood that the test pattern *x* belongs to the class *c*; larger *f*_*c*_(*x*) becomes, more likely the test pattern *x* belongs to the class *c*. Thus classification is done by comparing the values of the discriminant functions of all classes. For example, *f*_normal_(*x*) is larger than *f*_abnormal_(*x*) for *x*, the test pattern *x* is classified into “normal.”

Before using the discriminant function method, we need to specify the type of discriminant function. The most reasonable and straightforward definition of the discriminant function is class likelihood *p*(*x* | *c*), which is a probabilistic density function showing the probability (density) that the pattern *x* appears in the class *c*. This is closely related to Bayes classifier, which is an optimal classifier with the minimum misclassification risk. Similarly, many discriminant function methods are closely related to statistics and thus are called statistical pattern recognition (Jain *et al*. 2000).

Unfortunately, the use of class likelihood *p*(*x* | *c*) as a discriminant function is not easy. In fact, we do not know *p*(*x* | *c*) in advance. In addition, the estimation of *p*(*x* | *c*) is a well-known challenging problem in the area of statistics. Especially, when the number of training patterns is limited, it is impossible to estimate *p*(*x* | *c*) accurately. Consequently, we often assume that *p*(*x* | *c*) is a Gaussian function. Since a Gaussian distribution is specified just by its mean and covariance, the estimation of *p*(*x* | *c*) as a Gaussian function is easier than an arbitrary function. However, we should not forget the fact that even the estimation of the mean and covariance is difficult when the dimensionality of the feature vector *x* is large. If the number of training patterns are not far larger than the dimensionality, the estimated mean and the covariance may be unreliable. For example, it is difficult to estimate the shape of a 100-dimensional Gaussian distribution with five training patterns. This is so-called the Hughes phenomenon and a kind of “curse of dimensionality.”

### Anticipated difficulties on bioimage recognition tasks

In both of the nearest neighbor methods and discriminant function methods, it was emphasized that the number of training patterns should be sufficiently large for better classification performance. However, this requirement is too severe for biological image recognition because it is often impractical to collect a huge number of biological images as training patterns. A possible remedy for this situation is to use a lower-dimensional feature. Under a lower dimensionality, even a small number of patterns will show their true distribution. For example, it may be better to use the height and the width of a person's silhouette than an entire silhouette image for a healthy and unhealthy classification. Projection to a low-dimensional subspace (see Table 8) is also effective.

Another anticipated difficulty in bioimage recognition is the quality of the ground-truth. For example, it will be difficult to attach the perfect ground-truth “healthy” and “unhealthy” to each cell image even by a careful human inspection. In fact, there might be an intermediate cell image between “healthy” and “unhealthy.” A possible remedy is to introduce an option of “rejection.” For an ambiguous pattern, we stop classifying it into one of two classes. Instead, we give up classifying it. Another remedy is to introduce a weight for each decision-making. For example, in most diagnosis applications, misrecognition of an “unhealthy” person as “healthy” is a more severe mistake than misrecognition of a “healthy” person as “unhealthy.” Thus by weighing (i.e., penalizing) the decision on “healthy” decision larger than “unhealthy” decision, we can decrease the risk of misrecognition of “unhealthy” as “healthy”. A more essential remedy is to use a classifier robust to outliers. For example, k-nearest neighbor (k-NN) classifier is more robust than 1-nearest neighbor (1-NN) classifier. The k-NN classifier is robust because it takes the majority class from the *k* classes by *k* nearest neighbors.

As noted in Introduction with the example of leaf-type recognition, we may encounter the case where no predefined class exists in bioimage recognition tasks. In this case we need to start from defining classes in some way. Clustering is a reasonable tool for this because we can understand how entire patterns form clusters and then attach a class label to each cluster.

## Conclusion

Image processing and pattern recognition techniques are helpful to analyze bioimages. Since a huge number of techniques have been proposed, the choice of appropriate technique for a specific task is important. For example, there are many binarization techniques with different properties and therefore we need to understand what the best binarization technique for the task is. This paper can be used for a brief guide for helping the choice.

As emphasized, bioimages are a very difficult target even for state-of-the-art image processing and pattern recognition techniques. Thus, for a specific task, we may need to develop a new technique. This will be possible by a collaboration of biologists and specialists of image processing and pattern recognition with enough discussion. On the other hand, a task can be solved easily by an existing technique or a combination of existing techniques. Even in this case, it is worth discussing with an image processing specialist because she/he will help to choose appropriate techniques.

Like biology, research on image processing and pattern recognition continues steadily and will make further progress in accuracy, robustness, versatility, usability, computational efficiency, etc. Many biological tasks can use future image processing techniques for fully automatic image analysis. They also can use future (or even present) pattern recognition techniques for proving empirically known biological facts and discovering new biological facts. Again, for continued progress, mutual collaboration between biologists and image processing specialists is very important.

## Footnotes

1 Currently, the whole content is freely downloadable from the authors' website: http://homepages.inf.ed.ac.uk/rbf/BOOKS/BANDB/bandb.htm
